# The S128N mutation combined with an additional potential *N*-linked glycosylation site at residue 133 in hemagglutinin affects the antigenicity of the human H7N9 virus

**DOI:** 10.1038/emi.2016.66

**Published:** 2016-07-06

**Authors:** Wanli Liu, Tian Bai, Jinlei Guo, Xinlan Li, Lei Yang, Xiaojun Wang, Junfeng Guo, Xin Ma, Xiyan Li, Hongbin Liu, Jianfang Zhou, Dayan Wang, Yue-Long Shu

**Affiliations:** 1 The Center for Disease Control and Prevention, Urumqi, Xinjiang Uygur Autonomous Region 830001, China; 2 National Institute for Viral Disease Control and Prevention, Collaboration Innovation Center for Diagnosis and Treatment of Infectious Diseases, Chinese Center for Disease Control and Prevention, Key Laboratory for Medical Virology, National Health and Family Planning Commission, Beijing 102206, China

**Dear Editor,**

It has been well established that hemagglutinin (HA) is the critical viral target glycoprotein for host immunity^1^ and that the influenza virus can escape the pre-existing antiviral immune responses by changing its antigenicity through a process known as antigenic drift.^2, 3^ A computational analysis of the HA antigenic epitopes of avian and human H7N9 viruses circulating in 2013–2014^4^ has revealed that six of nine sites displayed a <95% conservation of location in the inferred epitopes. The data indicated the H7N9 virus might have already experienced gradual herd immunity-derived evolutionary pressure via natural infection. A glycosylation motif of N-X-T/S at site 133 (H3 numbering) has emerged in H7 subtype viruses, including some H7N9 viruses.^4^ A recent study identified an *N*-glycosylation site (NGS) at residue 133 generated by the presence of a threonine at position 135 (135T) in HA of the A/Netherlands/219/2003 (H7N7) pseudovirus and the mutant A/Shanghai/2/2013 (H7N9) pseudovirus.^5^ Furthermore, the appearance of 135T, rather than the alanine normally found in HA (135A), detected in the H7N9 vaccine strain significantly reduced HA reactivity to neutralizing monoclonal antibodies targeting the HA antigenic site A.^5^ Here, we report a human H7N9 virus with a reduced reaction to reference strain A/Anhui/1/2013 (AH1)-immunized serum. The amino acid substitutions in HA that contribute to the alterations in antigenicity were identified in this study.

On 29 July 2014, a 66-year-old woman with a history of exposure to live poultry was diagnosed with an H7N9 infection in the Xinjiang uyghur autonomous region. The clinical symptoms were present on 13 July 2014 and the patient died on 3 August 2014. An H7N9 influenza virus strain was isolated from the patient's respiratory clinical sample and denoted as A/Xinjiang/73030/2014 (XJ73030), and a virus strain named A/Environment/Xinjiang/73033/2014 (Ev73033, H7N9) was also isolated from the epidemiologically linked live poultry market. We performed antigenic analysis among these two viruses and other H7N9 viruses isolated in Mainland China in 2014 via the standard one-way hemagglutination inhibition assay (HI) using 0.5% turkey red blood cells in Biosafety level 3 (BSL-3) laboratory.^6^ The ferret antiserum against A/Anhui/1/2013 (AH1, the H7N9 vaccine candidate) was used. All tested H7N9 virus strains reacted well with vaccine strain AH1-immunized serum except for XJ73030, which exhibited a fourfold lower titer, indicating altered antigenicity for this strain (Supplementary Table S1). Sequencing results revealed four mutations, including N47H, S128N, A135T and L177I, in the human isolate XJ73030. The N47H, S128N and L177I mutations were also detected in the Ev73033 strain. Deep sequencing in the original respiratory clinical sample of the patient revealed similar mutations, although the T substitution at 135 only accounted for 8.1% of the mutations. Mutations were mapped according to the published structure of H7N9 HA protein^7^ (Supplementary Figure S1). The additional potential NGS at 133 as a result of the A135T mutation was located in the inferred antigenic site A around the globular head of HA, while site 135 was also located in the receptor-binding domain. Sites 47 and 128 were on the inferred epitopes C and B, whereas site 177 was not observed in the surface.

On the basis of HA sequences from 208 H7N9 strains in the global initiative on sharing all influenza data (GISAID) as of 19 September 2014, mutations in HA have been detected in 22 human or environmental H7N9 viruses (Supplementary Table S2). The L177I substitution was the most frequently occurring mutation, and the S128N mutation was only detected in one environmental virus. The potential NGS at site 133 as a result of S or T substitution at site 135 was detected in six H7N9 isolates. No N47H substitution was detected before our identification of the XJ isolates. To further explore the underlying mechanisms contributing to the reactivity of H7N9 to AH1-antiserum, nine H7N9 isolates possessing the single mutations of S128N, A135T or L177I were selected to perform antigenic analysis (Supplementary Table S3). The strains containing the A135T mutation alone showed a twofold decreased HI titer to A/Anhui/1/2013 immunized serum, whereas other strains showed a high degree of antigenicity to the reference virus, which indicated that the reduced antigenic reaction of XJ73030 might be caused by the combined effects of multiple mutations. Next, we perform another antigenic analysis by using seven reassortants bearing single or multiple mutations using the 8-plasmid reverse genetic technique which were rescued as previously described.^8^ The viruses were 6:2 reassortants consisting of the A/Puerto Rico/8/34 (H1N1) backbone with the *HA* and *neuraminidase* genes from AH1. In contrast with the human isolate XJ73030, we mutated A to S at 135 to evaluate the effect of NGS irrespective of an S or T mutation on altering the reactions with AH1 antiserum. We generated a strain with the combination of 133NGS and S128N, as mutations in antigenic epitopes A and B were relatively more frequent than the other three epitopes in the H7 Eurasian lineage^4^ and a slightly reduced titer was observed in the wild-type H7N9 virus containing 133NGS alone (Supplementary Table S3). Triple mutant reassortant viruses were generated by adding L177I or N47H mutations based on the double combination (133NGS+S128N+L177I, 133NGS+S128N+N47H). Two independent experiments were performed in this antigenic analysis. The HI titers of two testing results were same indicating good intra-laboratory reproducibility in this study. Similar to the results for nine wild-type H7N9 viruses bearing single mutations of S128N, A135T and L177I, the mutant virus with 133NGS alone showed a twofold reduction in HI titer, whereas other viruses with single mutations were antigenically similar to AH1 (Table 1). However, a fourfold reduced reaction to AH1 antiserum was observed in virus samples with a combination of 133NGS and S128N (Table 1), indicated that these mutations contributed to the reduced antigenic response to serum against AH1. Furthermore, the mutations of L177I and N47H in combination with 133NGS or S128N seem to have the opposite effect, i.e., increased twofold sensitivity to AH1 ferret serum comparing with the combination of 133NGS and S128N (Table 1). It is possible that the reactivity of antibodies targeting 133NGS and S128N might be attenuated by the effect of antibodies against L177I or N47H. However, as the result was detected by using a polyclone ferret antiserum against AH1 virus, the mechanism needs to be further characterized by a panel of monoclonal antibodies aimed at these sites.

In this study, we first identified a human H7N9 antigenic variant with four mutations in HA and addressed a combination of S128N and 133NGS contributed to the reduced antigenic response to the antiserum against AH1. This suggests that the immune response to antigenic sites A and B elicited by candidate vaccine viruses may not contribute to protection against H7 viruses that are glycosylated at site 133 and mutated from S to N at site 128. Although a fourfold or less titer in a one-way HI test has remained a marker of lower risk when assessing the antigenic relationship to vaccine candidates, this was the first time that an H7N9 virus was observed to have a reduced HI titer to a reference strain. Thus, this finding could be a warning indicating that the H7N9 virus has already experienced herd immunity pressure and that the emergence of additional antigenic drift in H7N9 viruses cannot be ignored. Through our surveillance database of the H7N9 virus, S or T substitutions at site 135 have been detected in human H7N9 strains since 2013. We found that the presence of 135T or 135S showed comparable effects on the antigenicity of the H7N9 virus suggesting that the effects might be associated with the appearance of *N*-glycosylation at site 133. In addition, the A135T mutation has been reported as a molecular marker for mammalian adaptation and virulence in H10N8 influenza virus.^9^ Therefore, enhanced surveillance of the observed mutations is critical for the selection and development of a H7N9 vaccine.

## Figures and Tables

**Table 1:** Hemagglutination inhibition reactions of reassortant viruses containing single or multiple mutations

**Reference virus**	**Pass.**	**Subtype**	**Ferret antisera**
			**A/Anhui/1/2013**
A/Anhui/1/2013 (reassortant virus)	E4	H7N9	320
			
*Mutated viruses*			
S128N	E3	H7N9	320
L177I	E3	H7N9	320
N47H	E3	H7N9	320
133NGS	E3	H7N9	160
133NGS+S128N	E2	H7N9	80
133NGS+S128N+L177I	E2	H7N9	160
133NGS+S128N+N47H	E2	H7N9	160

Abbreviation: Hemagglutination inhibition assay, HI. Two independent experiments were performed for the seven reassortant viruses in this study. HI titers of two testing results were same indicating good inner laboratory reproducibility of antigenic analysis in this study.
