# Supplementary material for: The S128N mutation combined with an additional potential N-linked glycosylation site at residue 133 in hemagglutinin affects the antigenicity of the human H7N9 virus
Source: Emerg Microbes Infect. 2016 Jul 6;5(7):e66–. doi: 10.1038/emi.2016.66 (PMC4972904; doi:10.1038/emi.2016.66)
Supplement: Supplementary Table S1 [file emi201666x1.pdf]

Reference viruses	Pass.	Collection date	Subtype	Ferret antisera
				**A/Anhui/1/2013**
A/Anhui/1/2013	E2		H7N9	160
**Testing viruses**				
A/Fujian/16/2014	E1	2014/3/28	H7N9	160
A/Zhejiang/24/2014	E1	2014/1/24	H7N9	320
A/Zhejiang/23/2014	E1	2014/2/9	H7N9	320
A/Zhejiang/22/2014	E1	2014/2/8	H7N9	80
A/Zhejiang/21/2014	E1	2014/2/10	H7N9	160
A/Zhejiang/20/2014	E1	2014/2/3	H7N9	160
A/Zhejiang/19/2014	E1	2014/1/23	H7N9	160
A/Zhejiang/18/2014	E1	2014/2/5	H7N9	160
A/Zhejiang/17/2014	E1	2014/2/3	H7N9	80
A/Zhejiang/16/2014	E1	2014/2/3	H7N9	160
A/Zhejiang/15/2014	E1	2014/1/25	H7N9	320
A/Zhejiang/14/2014	E1	2014/1/14	H7N9	160
A/Zhejiang/13/2014	E1	2014/1/27	H7N9	160
A/Fujian/15/2014	E1	2014/2/26	H7N9	160
A/Jiangxi/27569/2014	E1	2014/4/21	H7N9	160
A/Hunan/26937/2014	E1	2014/4/19	H7N9	160
A/Environment/Xinjiang/73033/2014 (Ev73033)	E1	2014/7/25	H7N9	80
A/Xinjiang/73030/2014 (XJ73030)	E1	2014/7/25	H7N9	40

Supplemental Table 1. Hemagglutination inhibition reactions of selected wild type H7N9 viruses
