# Supplementary material for: The S128N mutation combined with an additional potential N-linked glycosylation site at residue 133 in hemagglutinin affects the antigenicity of the human H7N9 virus
Source: Emerg Microbes Infect. 2016 Jul 6;5(7):e66–. doi: 10.1038/emi.2016.66 (PMC4972904; doi:10.1038/emi.2016.66)
Supplement: Supplementary Table S2 [file emi201666x2.pdf]

Virus	Amino acid substitutions (H3 numbering)																
	47	57	59	82	84	114	128	135	150	173	177	215	266	274	276	284	3
/2013	N	R	T	F	A	E	S	A	E	K	L	P	M	V	N	S	
05/2013	.	.	.	.	.	.	.	A/S	.	.	.	.	.	.	.	.	
nent/Jiangsu/03137/2013	.	.	.	.	.	.	.	.	.	.	I	.	.	.	.	.	
nent/Guangdong/25003/2013	.	.	.	.	.	.	.	.	D	.	I	.	.	.	.	.	
nent/Guangdong/02621/2013	.	K	.	.	.	.	N	.	.	.	.	.	.	.	.	.	
ong/02124/2014	.	K	.	.	.	.	.	.	D	.	I	.	.	.	.	.	
/07803/2014	.	.	.	.	.	.	.	A/T	.	.	.	.	.	.	.	.	
/17/2014	.	K	.	.	.	.	.	T	.	.	.	L	.	.	.	.	
/22/2014	.	K	.	.	.	.	.	T	.	.	.	.	.	.	.	.	
7833/2014	.	K	.	.	.	.	.	S	.	.	.	.	.	.	.	.	
ong/0012/2014	.	K	.	.	.	.	.	.	D	.	I	.	.	.	.	.	
09387/2014	.	.	I	L	.	.	.	.	.	R	I	.	I	.	.	.	
6938/2014	.	K	.	.	.	K	.	.	.	.	I	.	.	.	D	.	
6937/2014	.	K	.	.	.	K	.	.	.	.	I	.	.	.	D	.	
a/228/2014	.	K	.	.	.	K	.	.	.	.	I	.	.	.	D	.	
ong/4495/2014	.	K	.	.	.	K	.	.	.	.	I	.	.	.	D	.	
ong/5581/2014	.	K	.	.	T	K	.	.	.	.	I	.	.	.	D	N	
ong/5731/2014	.	K	.	.	.	K	.	.	.	.	I	.	.	I	G	.	
ong/8122430/2014	.	K	.	.	.	K	.	.	.	.	I	.	.	.	D	.	
1/2014	.	.	.	.	.	.	.	.	.	.	I	.	.	.	.	.	
2/2014	.	.	.	.	.	.	.	.	.	.	I	.	.	.	.	.	
ment/Xinjiang/73033/2014	H	.	.	.	.	.	N	.	.	.	I	.	.	.	.	.	
/73030/2014 (XJ73030)	H	.	.	.	.	.	N	T	.	.	I	.	.	.	.	.	
/73030/2014 Original clinical sample)	H	.	.	.	.	.	N	A(91.7%)/T(8.1%)*	.	.	I	.	.	.	.	.	

*Sequenced by deep sequencing, the percentages were calculated using the number of reads.

Supplemental Table S2. Mutations identified on HA protein of H7N9 viruses
