# Supplementary material for: The S128N mutation combined with an additional potential N-linked glycosylation site at residue 133 in hemagglutinin affects the antigenicity of the human H7N9 virus
Source: Emerg Microbes Infect. 2016 Jul 6;5(7):e66–. doi: 10.1038/emi.2016.66 (PMC4972904; doi:10.1038/emi.2016.66)
Supplement: Supplementary Table S3 [file emi201666x3.pdf]

Reference antigens	Pass.	Subtype	Ferret antisera<br>A/Anhui/1/2013	Mutations in HA
A/Anhui/1/2013	E2	H7N9	80	
**Testing viruses**				
A/Env/Guangdong/02621/2013	E1	H7N9	80	S128N
A/Zhejiang/07803/2014	E1	H7N9	40	A135A/T
A/Env/Jiangsu/03137/2013	E1	H7N9	80	L177I
A/Guangdong/02124/2014	E1	H7N9	80	L177I
A/Guangdong/0012/2014	E1	H7N9	80	L177I
A/Jiangsu/09387/2014	E1	H7N9	80	L177I
A/Environmental/Guangdong/25003/2013	E1	H7N9	160	L177I
A/Hunan/26937/2014	E1	H7N9	160	L177I
A/Hunan/26938/2014	E1	H7N9	80	L177I

Supplemental Table S3. Antigenic analysis on H7N9 virus with similar single mutations with A/Xinjiang/73030/2014
